# Chemiluminescent polymeric nanoprobes for tumor diagnosis: A mini review

**DOI:** 10.3389/fchem.2022.1106791

**Published:** 2023-01-09

**Authors:** Xiaoyan Zhang, Cong Li, Wenjuan Chen, Guanhua Wang, Huiru Zou, Hao Liu

**Affiliations:** ^1^ Central Laboratory, Tianjin Stomatological Hospital, School of Medicine, Nankai University, Tianjin, China; ^2^ Tianjin Key Laboratory of Oral and Maxillofacial Function Reconstruction, Tianjin, China

**Keywords:** chemiluminescence, tumor diagnosis, luminol, peroxyoxalates, 1,2-dioxetanes

## Abstract

Chemiluminescence (CL), a distinct luminescent process by taking advantage of chemical reactions rather than external light source, has recently attracted considerable research interests due to its high sensitivity and low background signal. The sensitivity and specificity of chemiluminescent signals in complex tumor microenvironment provide a sound basis for accurate detection of tumors. Various chemiluminescent nanoprobes with superior performance have been obtained by structural modification of chemiluminescent units or introduction of fluorescent dyes. In this review, we focused on the recent progress of chemiluminescent polymeric systems based on various chromophore substrates, including luminol, peroxyoxalates, 1, 2-dioxetanes and their derivatives for tumor detecting. And we also emphasized the design strategies, mechanisms and diagnostic applications of representative chemiluminescent polymeric nanoprobes. Finally, the critical challenges and perspectives of chemiluminescent systems usage in tumor diagnosis were also discussed.

## Introduction

Optical imaging plays a vital role in early diagnosis and treatment of diseases. It not only visualizes the detection of lesion sites, but also improves the accuracy of disease treatment (Thomas, 2015). However, due to tissue self-illumination, low tissue penetration depth and less noise interference, conventional optical imaging limits its application in living organisms (Li and Pu, 2019). Chemiluminescence refers to the process in which luminescence is generated through chemical reactions without external light source or other energy. In brief, chemical substances are oxidized into unstable high-energy intermediates, which subsequently disintegrate to emit light or transfer energy to surrounding fluorophores (Vacher et al., 2018). Chemiluminescence possesses the advantages of high sensitivity, deep tissue penetration depth, without external light source required, and high signal-to-background ratio, which provides new methods and ideas for the further development of optical imaging technology (Gnaim et al., 2018).

Chemiluminescence can be classified into two types, namely direct chemiluminescence and indirect chemiluminescence according to the energy conversion principle of luminescence (Yan et al., 2019). Direct chemiluminescence refers to the oxidation of a chemiluminescent substrate to form an excited-state intermediate with high energy, which then returns to the ground state to release photons, followed by the emission of light. The most representative is the luminol chemiluminescent system (Li et al., 2022). Indirect chemiluminescence generally involes chemiluminescence resonance energy transfer (CRET) process. 1, 2-dioxetane derivatives or peroxyoxalates are widely used as typical indirect CL systems for biomedical assays (Su et al., 2019; Tzani et al., 2021).

Despite the rapid development of chemiluminescence, the application of chemiluminescent substrate in biomedical fields is still limited compared to fluorescence due to its hydrophobicity, weak signal, and fast decay (Miao and Pu, 2018). To overcome these shortcomings, chemiluminescence systems combined with nanotechnology or different modifications have emerged and aroused the wide interest (Tiwari and Dhoble, 2018). In recent years, polymeric nanoparticles have attracted extensive attention due to their high brightness and high quantum yield, as well as their low toxicity, good biocompatibility and various synthesis methods (Kaeser and Schenning, 2010). This review aims to sum up recent advances in chemiluminescent polymeric nanoprobes based on three chromophore substrates, including luminol, peroxyoxalates, and 1, 2-dioxetanes. Moreover, the design strategies and luminescence mechanisms of different chemiluminescent polymeric nanoprobes are discussed, and their applications in tumor diagnosis are further elaborated.

### Luminol-based chemiluminescent polymeric nanoprobes

Luminol (5-amino-2, 3-dihydrophthalazine-1, 4-dione) and its derivatives are currently the most classical chemiluminescent reagents. In 1928, Albrecht (1928) discovered that luminol could emit CL when reacting with oxidizing agents such as hydrogen peroxide (H_2_O_2_) in alkaline media. Despite its low detection limit and good selectivity, the luminol chemiluminescent probe still suffers from a short emission wavelength (∼425 nm) and limited tissue penetration depth, hindering the application of this system in bioimaging *in vivo* (Zhang et al., 2013). The chemiluminescent properties of luminol itself are susceptible to external factors, therefore, nanomaterials with different catalytic properties are often used to modulate the performance of this chemiluminescent system. Graphene oxide was utilized to amplify the CL signal of luminol (Yang et al., 2014). Huang et al. (2006) demonstrated effective CRET between luminol and CdTe quantum dots and the red shift of the luminescence wavelength to the quantum dots in the luminol/H_2_O_2_ CL system. In addition, the researchers obtained luminol-based chemiluminescenct polymeric systems with improved properties by modifying luminol or linking it with different fluorescent dyes.

**FIGURE 1:**
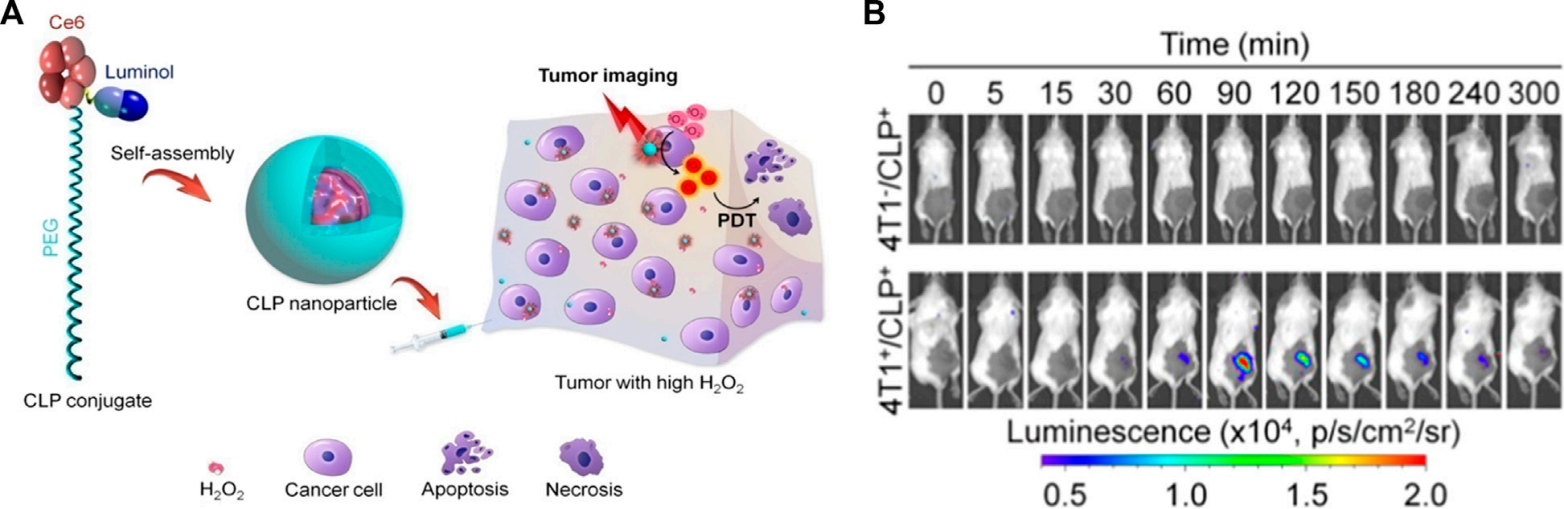
**(A)** Schematic illustration of CL imaging of tumors with high H_2_O_2_ and H_2_O_2_-mediated photodynamic therapy using CLP nanoparticles. **(B)** Time-dependent *in vivo* CL imaging of healthy mice and 4T1 tumor-bearing mice after local injection of CLP nanoparticles (An et al., 2020).

Reactive oxygen species are critical for cancer development, progression, and metastasis, thus early detection of ROS within cancer cells is crucial for the monitoring and management of cancers. Abnormal superabundant generation of H_2_O_2_, a major ROS, has been closely associated with cancer development (Trachootham et al., 2009). An et al. (2020) utilized luminol and poly (ethylene glycol) to simultaneously couple chlorin e6 to form an amphiphilic conjugate (defined as CLP), followed by the self-assembly of CLP to form luminescent nanoparticles. These nanoparticles with core-shell nanostructures could be activated by H_2_O_2_, leading to CL imaging and *in situ* photodynamic therapy (PDT) of tumors with high expression of H_2_O_2_, shown in Figures 1A, B. Nan et al. (2019) designed a polycarbonate copolymer (PMPC-ONA) micelle decorated with benzyl alcohol and then luminol, fluorophore and heme were encapsulated into the micelle to form the L/H/S@PMPC-ONA nanoprobe for H_2_O_2_ imaging *in vivo*. Once H_2_O_2_ encountered the heme in the nanoparticles, ROS were generated, and then luminol would be excited to trigger chemiluminescence, while the stability of the nanoparticles decreases, thus releasing the fluorescent indicator to detect H_2_O_2_. Chemiluminescence exhibits the potential for ROS detection since no excitation light source needs to be involved and the relationship between light emission and analyte concentration is explicit. Lee et al. (2016) designed hydrogen peroxide responsive hybrid nanoparticles (HNPs) consisting of the PEGylated QDs and a luminol derivative (L012) as the CL agent. The energy transduction owning to the reaction between L012 and H_2_O_2_ enabled HNPs to achieve light production. Furthermore, as a promising diagnostic agent for cancer, HNPs displayed non-invasive near-infrared (NIR) imaging of H_2_O_2_ *in vivo* without background interference. Endogenous photoactivation is also of great importance for the diagnosis and treatment of tumors. Mao et al. (2021) constructed two gold nanoparticles (tAuNP and makuNP) by modifying 2, 5-diphenyltetrazole and methacrylic acid on the surface of gold nanoparticles for tumor imaging and therapy. The mAuNPs were absorbed with luminol to form self-illuminating mAuNP/Lu nanoparticles. Owning to the tetrazole/alkene cycloaddition, H_2_O_2_-initiated mAuNP/Lu nanoparticles could specifically crosslink with tAuNP nanoparticles to generate large particle aggregates. Subsequently, these aggregates contributed to strong CL effect catalyzed by H_2_O_2_ in tumor microenvironment, leading to strengthened uptake and retention of AuNPs.

The specific identification of tumor markers in the blood and tissues of cancer patients is significant for early cancer diagnosis (Freedland, 2011). Yan et al. (2022) constructed a dual-carrier CL sensor that recognized tumor markers through an enzyme digestion CL signal amplification strategy. This sensor underwent aggregation and identified target markers, and then strong CL signals were collected under luminol catalysis reaction. Zhang et al. (2016) designed a novel signal enhancement strategy in which CRET occurred between reduced graphene oxide (rGO) as an energy acceptor and luminol as a donor. And the probe provided a more sensitive signal amplification strategy. Additionally, Ag-C_3_N_4_ nanosheet loaded with luminol capped AuNPs nanocomposite was developed as the electrochemiluminescence (ECL) signal nanoprobe for early tumor diagnosis (Liu et al., 2021). The CL signal could be obviously enhanced by cells in luminol-H_2_O_2_ system, and the signal intensity and the cell concentration were positively correlated. Based on the above phenomena, Ding et al. (2020) designed a new cell-assisted enhanced CL strategy to identify tumor cells rapidly. The above probes provided highly accurate and quantitative analysis of tumor markers in complex biological samples, showing great potential for clinical application.

### Peroxyoxalate-based chemiluminescent polymeric nanoprobes

The reaction of anthracene, 9, 10-diphenylanthracene and N-methylacridine with hydrogen peroxide and oxalyl chloride can produce a “blue-white light,” which is known as peroxyoxalate chemiluminescence (PO-CL), firstly discovered by Chandross (1963). PO-CL modulates the emission wavelength by adding different fluorophores to the reaction system, rather than modifying the CL molecule. PO-CL systems belong to indirect chemiluminescence, generally consists of oxalate, oxidant (usually hydrogen peroxide) and suitable dye. The formation of high-energy 1, 2-dioxadione intermediates is a key step leading to chemiluminescence emission (Delafresnaye et al., 2020). The three most commonly used oxalate eaters are TCPO (bis (2, 4, 6-trichlorophenyl) oxalate), CPPO (bis (2, 4, 5-trichlorophenyl-6-carbopentoxyphenyl) oxalate) and DNPO (bis (2, 4-dinitrophenyl) oxalate) (Lee et al., 2012). The detection of H_2_O_2_ in water, microorganisms in food, and blood samples in crime scenes all rely on the PO-CL reaction (García and Baeyens, 2000). Thanks to its high sensitivity, high quantum yield and long luminescence lifetime, peroxyoxalate-based chemiluminescence systems have been widely used in bioimaging and tumor therapy (Yang and Zhang, 2021).

PO-CL systems are usually designed as nanoparticles for *in vivo* bioimaging because of their susceptibility to decomposition when exposed to water. Shuhendler et al. (2014) developed a nanoparticle based on a dual-channel imaging function for imaging liver injury in mice, i.e., CRET-based peroxyoxalate chemiluminescence for H_2_O_2_ detection and fluorescence resonance energy transfer (FRET)-based fluorescence for ONOO^−^ and hypochlorite detection. The probe allowed for rapid and real-time direct assessment of acute hepatotoxicity. Zhen et al. (2016) constructed a H_2_O_2_-responsive chemiluminescent semiconducting polymer nanoparticle (SPN) using TCPO and applied it to the detection of endogenous H_2_O_2_ in peritonitis and neuroinflammation. With high brightness and proper near-infrared window, the SPN achieved ultrasensitive detection of H_2_O_2_. A PO-CL system based on CPPO with cascaded CRET and FRET processes was designed to permit near-infrared region II (NIR-II) chemiluminescence imaging in arthritic mice (Yang et al., 2020). The strategy avoided the loss of energy transfer as much as possible through the rational design of the probe.

Chemiluminescent polymeric nanoprobes that react with H_2_O_2_ within the tumor microenvironment may achieve accurate imaging of tumors. Yu et al. (2022) combined pluronic F-127 and polymer containing oxalate ester (POE) to form nanoparticles by means of hydrophilicity and hydrophobicity. The nanoparticles loaded anti-tumor drug could realize the tumor tracking by H_2_O_2_-related chemiluminescence and the tumor therapy by drug releasing, which possessed great potential for precise localization and efficient treatment of tumor. In addition, since tumor cells consume more glucose than normal tissues, the glucose level of tumor tissues ware also utilized to enable tumor detection and imaging (Li et al., 2019).

Apart from disease diagnosis related to hydrogen peroxide overproduction, the CL platform can be used to perform PDT, avoiding the limitations of external light sources and light penetration depth (Yuan et al., 2012). Excessive hydrogen peroxide is an intrinsic feature of tumor cells. Andrey et al. took advantage of this feature to designed and synthesised a dispersion composed of polyoxalate and tetramethylhematoporphyrin (TMHP). In the presence of TMHP, singlet oxygen (^1^O_2_) formation by reaction with endogenous H_2_O_2_ through PO-CL leaded to tumor cell elimination (Romanyuk et al., 2017). Wu et al. (2019) came up with a self-luminescing nanoreactor by coencapsulating CPPO, poly [(9,9′-dioctyl-2,7-divinylene-fluorenylene)-alt-2-methoxy-5-(2-ethyl-hexyloxy)-1,4-phenylene] (PFPV), and the photosensitizer tetraphenylporphyrin with polyethylene glycol-polycaprolactone (PEG-PCL) and folate-PEG-cholesterol. This novel system could achieve self-luminescence emission and singlet oxygen (^1^O_2_) production, which had important implications for imaging and treatment of cancer. Most conventional photosensitizers usually suffer from aggregation-caused quenching (ACQ) effects, resulting in reduced luminescence intensity and ROS generation (Li et al., 2021). The emergence of aggregation-induced emission luminogens (AIEgens) exhibiting enhanced fluorescence intensity and ROS production largely solves the above problems. Mao et al. developed a novel nanoparticle (C-TBD NPs) with chemiexcited far-red/NIR emission and ^1^O_2_ production capability using pluronic F127 and soybean oil co-loaded with CPPO and TBD, a photosensitizer with AIE properties. C-TBD NPs could emit chemiluminescence in response to hydrogen peroxide at the tumor site, thus enabling precise tumor tracking *in vivo*. In addition, C-TBD NPs also produced effective ^1^O_2_ to induce apoptosis of tumor cells (Mao et al., 2017).

Chemodynamic therapy (CDT), a new class of oncology therapeutic techniques based on the iron-based Fenton reaction, offers an alternative opportunity for cancer treatment (Yang et al., 2019). In order to enhance therapeutic efficiency and minimize side effects, the real-time monitoring of ROS production during CDT is extremely essential. However, CDT reagents that can emit ROS-associated signals are rare. Wang et al. (2019) constructed a semiconducting polymer nanoplatform containing CPPO, hemin and glucose oxidase (GOD). This ROS-dependent chemiluminescence of SPN allowed optical monitoring of intra-tumor ROS production during CDT. This nanoplatform represented the first intelligent strategy to enable chemiluminescence imaging for monitoring CDT, demonstrating great potential in assessing treatment responsiveness and predicting early treatment outcomes.

### 1, 2-dioxetane-based chemiluminescent polymeric nanoprobes

Chemiluminescent systems based on luminol and peroxyoxalates require the involvement of oxidants, making them often used for the detection of active species, which limits their scope of application. 1, 2-dioxetanes do not require the involvement of additional oxidants (hydrogen peroxide, oxygen, potassium permanganate, etc.), which simplifies the analytical process, improves detection sensitivity, and expands the field of chemiluminescence applications (Kopecky and Mumford, 1969; Schaap et al., 1989). Schaap’s group found that the deprotonation of the phenolic hydroxyl substituent could transform 1, 2-dioxetane into a superior luminescent intermediate in 1982 (Schaap and Gagnon, 1982). Subsequently, Schaap’s group further improved the thermal stability of 1, 2-dioxetane derivatives by introducing adamantane substituents (Schaap et al., 1987). However, previous 1, 2-dioxetane CL systems had a great tendency to be quenched by water, resulting in short emission wavelength and low luminescence intensity, which made it difficult to be applied *in vivo*.

To enhance the chemiluminescence intensity, the researchers have made numerous attempts, i.e., simple or complex modifications of the dioxetane scaffold: 1) the processing of dioxetane units into polymer monomers (Gnaim and Shabat, 2017). 2) the covalent binding of fluorescent dyes with higher fluorescence efficiency to Schaap’s 1, 2 dioxetanes (Matsumoto, 2004; Hananya et al., 2016). 3) the introduction of electron-withdrawing groups in the neighboring positions of phenoxy 1, 2-dioxetane (Eilon et al., 2018). Based on the above basic research, the researchers designed and constructed various chemiluminescent polymeric systems based on 1, 2-dioxetanes, which showed excellent performance in cancer detection.

**FIGURE 2:**
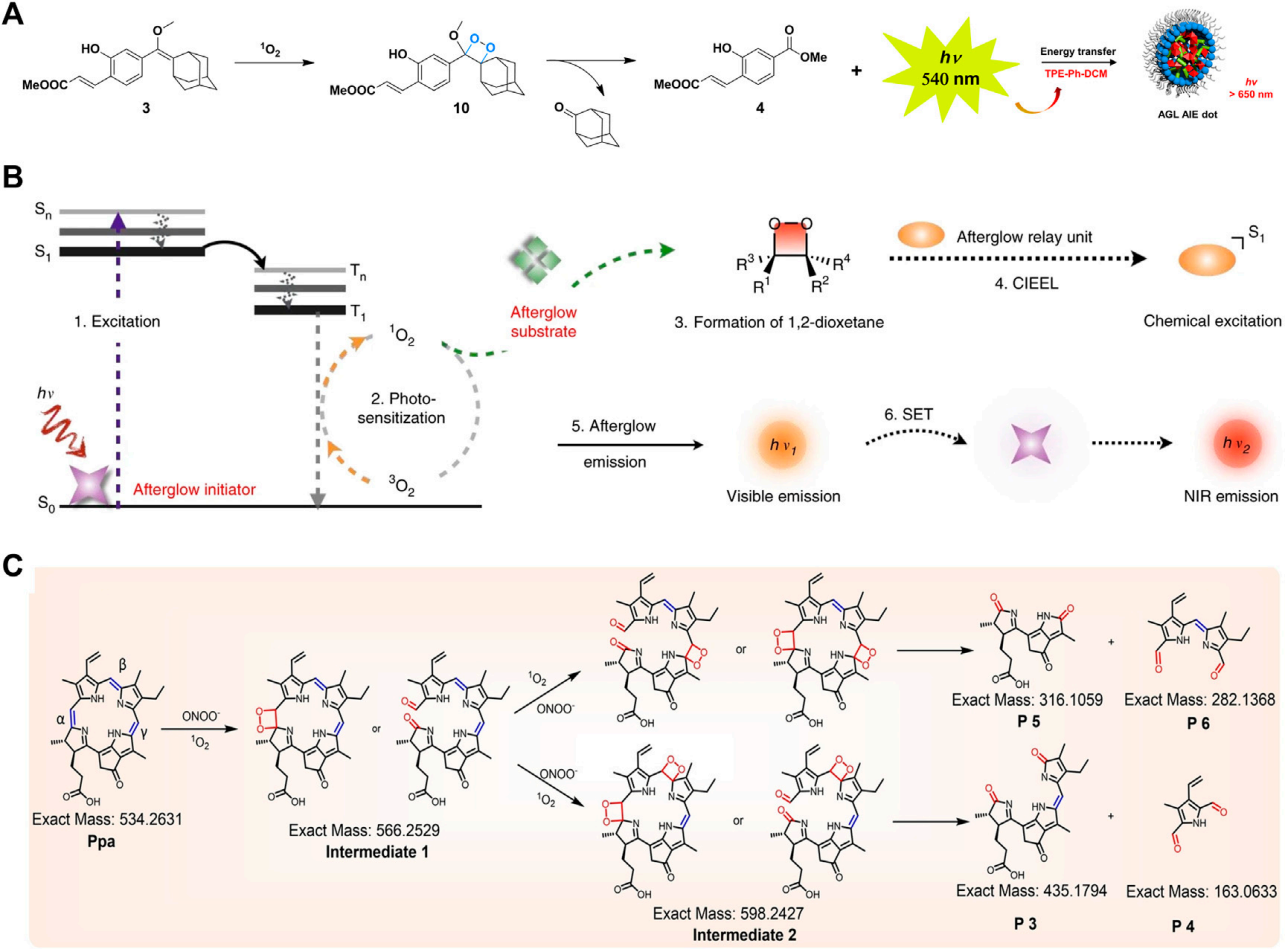
**(A)** Schematic diagram of the mechanism for NIR afterglow of the AGL AIE Dot (Ni et al., 2018). **(B)** Schematic diagram of the mechanism for intraparticle afterglow luminescence of ALNPs (Jiang et al., 2019). **(C)** Proposed mechanism for persistent luminescence of pheophorbide A (Duan et al., 2022).

Afterglow luminescence is a process of persistent luminescence after the cessation of light excitation, and afterglow imaging holds great promise in the biomedical fields. Ni et al. constructed a NIR afterglow luminescent nanoparticle (AGL AIE dots) with AIE characteristics by encapsulating the NIR emissive AIE molecule (TPE-Ph-DCM) and the enol ether precursor of Schaap’s 1, 2-dioxetane with Lipid-PEG_2000_ through nanoprecipitation. The AGL AIE dots finally emitted NIR afterglow luminescence through the generation of ^1^O_2_ by TPE-Ph-DCM, the formation and decomposition of dioxetane, the release of chemical energy, and the energy transfer to TPE-Ph-DCM, which lasted for more than 10 days after single light excitation through self-cycling luminescence mechanism. The afterglow luminescent signal of AGL AIE dots was extremely quenched in the liver, therefore, AGL AIE dots had great prospects in the application of accurate image-guided tumor surgery, shown in Figure 2A (Ni et al., 2018). Immunotherapy, an emerging cancer treatment in recent years, offers new treatment options for cancer patients, yet the response rate of patients in clinical applications has not been significant (Sambi et al., 2019). In order to improve the efficacy of immunotherapy, monitoring the immune responses of patients is inevitable. Cui et al. (2020) constructed semiconducting polymeric nanoreporters (SPNRs) by means of combining the semiconducting polymer and the dioxetane derivative. As the first reporter that could release chemiluminescent signals activated by superoxide anions, SPNRs could sensitively distinguish immune cells containing high O_2_-levels from other cells including cancer cells and normal cells, enabling real-time imaging of immune activation during cancer immunotherapy *in vivo*. The aforementioned PDT is a promising strategy for cancer therapy. Fan et al. (2021) designed nanoparticles that specifically respond to alkaline phosphatase overexpressed on hepatocellular carcinoma cells, ultimately producing ^1^O_2_ and NIR fluorescence for tumor diagnosis and treatment.

In addition to the direct addition of dioxetane units, some chemiluminescence systems can also emit chemiluminescence by forming unstable dioxetane intermediates in response to the tumor microenvironment. Miao et al. (2017) applied PEG-*b*-PPG-*b*-PEG coated poly (2methoxy-5-(2-ethylhexyloxy)-1, 4-phenylenevinylene) (MEHPPV) to prepare SPNs to amplify and redshift the afterglow effectively. Under the oxidation of PPV, SPNs generated unstable dioxolane intermediates, which slowly degraded into PPV aldehyde and emitted afterglow. This strategy could be applied to the detection of lymph nodes and tumors in living mice. Jiang et al. (2019) demonstrated an excellent method for converting ordinary fluorescent agents into afterglow luminescent nanoparticles (ALNPs) by forming unstable 1, 2-dioxetanes intermediates in order to detect tumors *in vivo* rapidly. This method consisted of the following steps: the photosensitizer absorbed light and converted it into ^1^O_2_, then the reactive molecule reacted with ^1^O_2_ to generate unstable 1, 2-dioxetane, and event ually the luminescence emitted by receiving the energy from 1, 2-dioxetane through semiconducting polymer, shown in Figure 2B. Lu et al. (2020) developed a novel chemiluminescent polymeric system (ultrathin MnOx-SPNs) with both CDT and pH responsiveness. The ^1^O_2_ produced by MnOx in response to the acidic tumor microenvironment reacted with SP, followed by forming thiophene-dioxetane intermediates and transferring energy to SPNs, leading to real-time monitoring of ^1^O_2_ generation and ratiometric CL/FL imaging for guiding cancer therapy. Duan et al. (2022) demonstrated persistent luminescence was detected from porphyrins after stopping the excitation light or reacting with peroxynitrite, verifying the successive oxidation of vinylene bonds may form unstable dioxetane intermediates. Such supramolecular probes could realize the light-triggered function conversion from photoacoustic imaging to persistent luminescence imaging, resulting in the successful implementation of image-guided tumor surgery, shown in Figure 2C. The mechanisms, advantages and disadvantages of the three chemiluminescent substrates were shown in Table 1.

**TABLE 1:** Overview of three chemiluminescent substrates.

Platform	Mechanism	Advantages	Disadvantages
Luminol	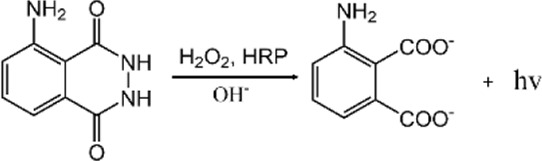	Good water solubility; Stable properties; High CL efficiency; Simple synthesis	Slow reaction rate; Short CL emission wavelength (425 nm); Shallow tissue penetration depths
Peroxyoxalate	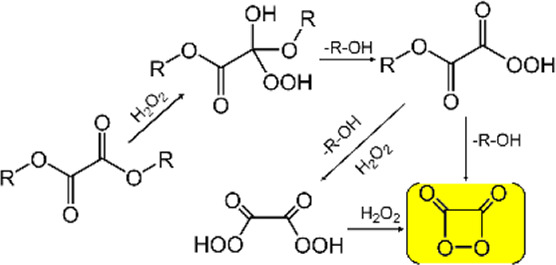	High sensitivity; High quantum efficiency (30% or higher for certain oxalate phenyl systems); Long luminescence lifetime	Poor compatibility with aqueous systems
1,2-dioxetane	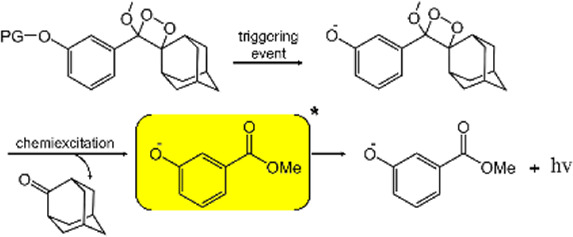	Deep penetration; Less light scattering; High sensitivity; Powerful luminescence intensity; Long half-lives	Complex synthesis; Short emission wavelength; Light instability

## Discussion

This article briefly reviewed recent developments of chemiluminescent polymeric nanosystems based on the chemiluminescence substrates including luminol, peroxyoxalates and 1, 2-dioxetanes. Moreover, luminescence mechanisms, design strategies and applications in biosensing and tumor diagnosis of chemiluminescent polymeric nanosystems were described in detail. An increasing number of studies have been performed to modify these three chemiluminescent substrates by direct or indirect means to improve luminescence efficiency and intensity for highly sensitive and precise quantitative analysis of solid tumors and even tumor markers. However, despite the remarkable progress in a wide range of fields, there still exist some problems that need to be solved and broken through: 1) Chemiluminescent systems based on 1, 2-dioxetanes have limited their biological applications due to their complex synthetic routes and short emission wavelengths. 2) Similar to water-soluble and lipid-soluble fluorescent dyes, the released dye tends to diffuse from the reaction site when a chemiluminescent probe is activated, making it more difficult to provide *in situ* information for imaging. The introduction of a second near-infrared (NIR-II) window (1,000–1,700 nm) into chemiluminescence imaging can achieve deeper penetration depths and higher signal-to-noise ratios, which is certainly helpful to solve the above issues. In addition, finding more stable fluorophores with higher antioxidant capacity and increasing the fluorescence quantum yield of fluorophores can maintain good efficiency of chemiluminescence, and the choice of enzymes-initiated chemiluminescence systems to avoid ROS/RNS oxidation is also an effective means. Over the past few years, chemiluminescent polymeric nanoprobes have developed significantly as imaging analytical tools for tumor diagnosis. In addition to tumor tissue imaging, the chemical flexibility of chemiluminescent polymeric nanoprobes holds great promise for their application in multifunctional therapeutic platforms.
